# Azithromycin added to hydroxychloroquine for patients admitted to intensive care due to coronavirus disease 2019 (COVID-19)—protocol of randomised controlled trial AZIQUINE-ICU

**DOI:** 10.1186/s13063-020-04566-x

**Published:** 2020-07-08

**Authors:** František Duška, Petr Waldauf, Milada Halačová, Václav Zvoníček, Jakub Bala, Martin Balík, Jan Beneš, Olga Klementová, Irena Kozáková, Viktor Kubricht, Anne Le Roy, Tomáš Vymazal, Veronika Řehořová, Vladimír Černý, on behalf of Czech Anaesthesia Clinical Trials and Audit Network

**Affiliations:** 1 Department of Anaesthesia and Intensive Care, Charles University, 3rd Faculty of Medicine and FNKV University Hospital, Prague, Czech Republic; 2 Masaryk University, Medical Faculty and U Svate Anny University Hospital, Brno, Czech Republic; 3 Na Bulovce Hospital, Prague, Czech Republic; 4 Charles University, 1st Faculty of Medicine and VFN University Hospital Prague, Prague, Czech Republic; 5 Charles University, Medical Faculty and University Hospital Plzen, Pilsen, Czech Republic; 6 Medical Faculty, Palacky University and Olomouc University Hospital, Olomouc, Czech Republic; 7 Charles University, 2nd Faculty of Medicine, Motol University Hospital, Prague, Czech Republic; 8 Czech Anaesthesia Clinical Trials and Audit Network and Department of Anaesthesia and Intensive Care, Masaryk’s Hospital, Ústí nad Labem, Czech Republic

**Keywords:** Novel coronavirus, COVID-19, SARS-CoV-2, Azithromycin, Hydroxychloroquine, Respiratory failure

## Abstract

**Background:**

Novel coronavirus SARS-CoV-2 is known to be susceptible in vitro to exposure to hydroxychloroquine and its effect has been found to be potentiated by azithromycin. We hypothesise that early administration of hydroxychloroquine alone or in combination with azithromycin can prevent respiratory deterioration in patients admitted to intensive care due to rapidly progressive COVID-19 infection.

**Methods:**

Design: Prospective, multi-centre, double-blind, randomised, controlled trial (RCT). Participants: Adult (> 18 years) within 24 h of admission to the intensive care unit with proven or suspected COVID-19 infection, whether or not mechanically ventilated. Exclusion criteria include duration symptoms of febrile disease for ≥ 1 week, treatment limitations in place or moribund patients, allergy or intolerance of any study treatment, and pregnancy. Interventions: Patients will be randomised in 1:1:1 ratio to receive Hydroxychloroquine 800 mg orally in two doses followed by 400 mg daily in two doses and azithromycin 500 mg orally in one dose followed by 250 mg in one dose for a total of 5 days (HC-A group) or hydroxychloroquine + placebo (HC group) or placebo + placebo (C-group) in addition to the best standard of care, which may evolve during the trial period but will not differ between groups. Primary outcome is the composite percentage of patients alive and not on end-of-life pathway who are free of mechanical ventilation at day 14. Secondary outcomes: The percentage of patients who were prevented from needing intubation until day 14, ICU length of stay, and mortality (in hospital) at day 28 and 90.

**Discussion:**

Although both investigational drugs are often administered off label to patients with severe COVID-19, at present, there is no data from RCTs on their safety and efficacy. In vitro and observational trial suggests their potential to limit viral replication and the damage to lungs as the most common reason for ICU admission. Therefore, patients most likely to benefit from the treatment are those with severe but early disease. This trial is designed and powered to investigate whether the treatment in this cohort of patients leads to improved clinical patient-centred outcomes, such as mechanical ventilation-free survival.

**Trial registration:**

Clinical trials.gov: NCT04339816 (Registered on 9 April 2020, amended on 22 June 2020); Eudra CT number: 2020-001456-18 (Registered on 29 March 2020).

## Administrative information

Note: the numbers in curly brackets in this protocol refer to SPIRIT checklist item numbers. The order of the items has been modified to group similar items (see http://www.equator-network.org/reporting-guidelines/spirit-2013-statement-defining-standard-protocol-items-for-clinical-trials/).

**Table:** 

Title {1}	Azithromycin added to hydroxychloroquine in patients admitted to intensive care due to coronavirus disease 2019 (COVID-19)—randomised controlled trial AZIQUINE-ICU
Trial registration {2a and 2b}.	Clinical trials.gov: NCT04339816 Eudra CT number: 2020-001456-18
Protocol version {3}	AZIQUINE-ICU 25032020 Version 1.5
Funding {4}	The trial is funded by Donatio Intensivistam Endowment Fund www.donatio-intensivistam.cz, VAT No 0907206 Registered under No N1795 by Municipal Court in Prague on 2 April 2020, trial drugs were provided as a kind gift of Zentiva, a.s. and a rapid grant from COVID-19 GAMA-2 scheme of Technology Agency of the Czech Republic
Author details {5a}	• František Duška, Milada Halačová, Petr Waldauf, Anne Le Roy, Irena Kozáková, Viktor Kubricht, Veronika Řehořová from Dept. of Anaesthesia and Intensive Care Charles University, 3rd Faculty of Medicine and FNKV University Hospital, Prague, Czech Republic • Jakub Bala, Na Bulovce Hospital, Prague, Czech Republic • Martin Balík, Charles University, 1st Faculty of Medicine and VFN University Hospital Prague, Czech Republic • Jan Beneš, Charles University, Medical Faculty and University Hospital Plzen, Czech Republic • Olga Klementová, Medical Faculty, Palacky University and Olomouc University Hospital, Czech Republic • Tomáš Vymazal, Charles University, 2nd Faculty of Medicine, Motol University Hospital, Prague, Czech Republic • Václav Zvoníček, Masaryk University, Medical Faculty and U Svate Anny University Hospital, Brno, Czech Republic • Vladimír Černý from Czech Anaesthesia Clinical Trials and Audit Network and Dept. of Anaesthesia and Intensive Care, Masaryk’s Hospital, Ústí nad Labem, Czech Republic
Name and contact information for the trial sponsor {5b}	Donatio Intensivistam Endowment Fund VAT No 0907206 Registered under No N1795 by Municipal Court in Prague on 2 April 2020. Address: Hornoměcholupská 40c/1640, 10200 Prague 15, Czech Republic www.donatio-intensivistam.cz, František Duška, MD, PhD, founder Email: frantisek.duska@lf3.cuni.cz Phone: +420608405541
Role of sponsor {5c}	This is an investigator-initiated study. Study funders had no role in the study design; collection, management, analysis, and interpretation of data; nor in writing of the report or the decision to submit the report for publication.

## Introduction

### Background and rationale {6a}

At the beginning of 2020, the novel coronavirus disease (COVID-19) began to spread from Asia to Europe and beyond forcing the World Health Organization to declare a global pandemic. Infected patients shed the virus for a median of 20 days [1]. Up to 10% of COVID-19 infected patients develop a severe form of disease requiring intensive care admission and some of them die. SARS-Cov2 is an encapsulated positive-strand RNA virus that uses ACE-2 of type 2 pneumocytes as binding sites. It has been hypothesised (Gattinoni 2020 ICM editorial) that initial hypoxemia caused by loss of primary injury to pulmonary vasculature leads to hyperventilation and patients self-inflicted lung injury predominantly in lung areas of increase stress and strain. In turn, later during the course of the disease, ARDS develops with a typical restrictive pattern of a stiff, wet and recruitable lung. At present, there is no evidence-based causative treatment of SARS-CoV-2 and there is a burning need of randomised controlled trials to find effective therapeutic strategies intervention.

Chloroquine has been used for malaria treatment and chemoprophylaxis, and hydroxychloroquine is used for the treatment of rheumatoid arthritis, systemic lupus erythematosus and porphyrias. Both drugs have in vitro activity against SARS-CoV, SARS-CoV-2 and other coronaviruses, with hydroxychloroquine having relatively higher potency against SARS-CoV-2 known to be susceptible in vitro to exposure to hydroxychloroquine [2–4]. At the moment, clinical trials are ongoing to test clinical efficacy in pre- and post-exposure prophylaxis in SARS-CoV-2. In one highly cited French non-randomised observational study by Gautret et al., a significant reduction of virus carriage has been observed in patients coincidentally treated by azithromycin in addition to hydroxychloroquine [5] as a part of initial empirical therapy of community-acquired pneumonia. Azithromycin is a macrolide antibiotic, which binds to the 50S subunit of the bacterial ribosome, thus inhibiting translation of mRNA. The positive-sense RNA viruses and indeed all genes defined as positive-sense can be directly accessed by host ribosomes to immediately form proteins and the effects of azithromycin on this process are not known. No data are available at present on the clinical efficacy of hydroxychloroquine alone or in combination with azithromycin, and it is likely that any treatment affecting virus replication would only be effective if administered early, before overt ARDS develops.

In the light of this, we designed a trial in which we test a hypothesis that early administration of hydroxychloroquine alone or in combination with azithromycin can prevent respiratory deterioration in patients admitted to intensive care due to rapidly progressive COVID-19 infection.

## Objectives {7}

The primary study objective is to test the hypothesis that early administration of combination therapy slows disease progression and improves mechanical ventilation-free survival.

The secondary (exploratory) study objective is to investigate whether the intervention decreases the viral load and influences oxygenation and mechanical feature of the lung.

SPIRIT guidance: Specific objectives or hypotheses.

## Trial design {8}

Prospective, randomised (1:1:1), placebo and active-comparator controlled, parallel group, double-blind exploratory trial.

## Methods: participants, interventions and outcomes

### Study setting {9}

This is a multi-centre trial conducted in intensive care units of 8 hospitals (6 university hospitals and 2 district general hospitals) in the Czech Republic. The updated list of centres including contact details of site investigators can be found in the Appendix.

### Eligibility criteria {10}

For participants, the following eligibility criteria were set:

#### Inclusion criteria

- Adult (> 18 years)
- Within 24 h of admission to the intensive care unit
- Proven or suspected COVID-19 infection

#### Exclusion criteria

- Symptoms of febrile disease for ≥ 1 week at enrolment
- Pregnancy or inability/unwillingness to perform pregnancy test at and 28 days after enrolment
- Treatment limitations in place or moribund patients
- Allergy, intolerance or contraindication to any study drug, such as long QT syndromes, myasthenia gravis, pre-existing maculopathy or retinopathy, allergies or known deficiency of glucose-6-phosphate dehydrogenase
- Known HIV positivity
- Significant liver disease such as cirrhosis Child-Pugh C or active hepatitis B or C
- Known stage IV chronic kidney disease, on dialysis at enrolment or imminent need of it
- Participation in another outcome-based interventional trial within the last 30 days
- Patients taking hydroxychloroquine for other indication than to treat COVID-19

Comments to the inclusion criteria: For the purpose of this study, intensive care unit is defined as a facility that allows continuous monitoring of vital functions and oxygen administration. In other cultural contexts, this could include also high dependency unit and intermediary care units or level 2 units.

It is expected that most patients will have rtPCR test known within 24 h of admission to hospital. Nonetheless, if this is not the case (e.g., due to overloaded lab facility, lack of supplies) it is possible to randomise a patient based on a strong clinical suspicion of SARS-Cov-2 infection. In the case COVID-19 is not confirmed in retrospect, experimental therapy is withdrawn, and the study subject is withdrawn from “per protocol” analysis of the primary and secondary outcomes but remains in an “intention-to-treat” cohort for the analysis of safety.

Eligibility criteria for study sites. Study sites were selected based on track-record of performing multi-centre interventional studies and willingness to participate.

### Who will take informed consent? {26a}

There is a three-step process of obtaining informed consent process compliant with Declaration of Helsinki (ver. 2008): (1) Patients with decision-making capacity will be asked by study physician to provide written prospective informed consent and consent for the use and storage of personal data as per Regulation (EU) 2016/679 General Data Protection Regulation (see Appendix). It is expected that a significant proportion of screened patients will lack the capacity to provide informed consent due to altered consciousness, respiratory distress, or sedation to facilitate mechanical ventilation. In this situation, (2) the deferred consent policy [6] will be applied in patients without capacity: patient’s legal representative will be sought to consent for the patient and if not available or approachable with reasonable effort, (3) the patient will be enrolled without consent. In the latter case, an independent physician will confirm in writing that the patient fulfils criteria and lack capacity. The patients themselves are approached to provide ongoing consent as soon as they regain capacity. They are given options to continue in the study, to withdraw with permission to use the data collected up to the point or to withdraw from the study and request deleting all data collected. This trial is fully compliant with both Declaration of Helsinki (ver. 2008) and Czech legislation. The family will be informed when practical about their relative’s enrolment into the trial based on the opinion of the independent physician and the family will be offered an information leaflet explaining the nature of the study. In the case they are of the opinion that the patient would not wish to participate in the trial, patients will be withdrawn. All serious adverse events that are suspected from being related to study interventions will be reposted to Research Ethics Board and regulatory authorities as per local legislation.

#### Item {26b}

Additional consent provisions for collection and use of participant data and biological specimens in ancillary studies, if applicable.

Additional consent provisions for collection and use of participant data and biological specimens is not applicable to our trial as no ancillary studies are planned.

## Interventions

### Explanation for the choice of comparators {6b}

At present, there is no effective drug to treat COVID-19 and hence best supportive care in addition to randomising patients into high quality RCTs is recommended by World Health Organization [7], Infectious Diseases Society of America [8], Surviving Sepsis Campaign Guidelines endorsed by European Society of Intensive Care Medicine [9], and Czech Society of Intensive Care Medicine (www.csim.cz). All patients will receive best supportive care that will be monitored, but not protocolized. It is recognised that the standard of care may substantially differ among study centres and that is why randomisation is stratified. Concomitant medication may include off label use of unproven treatments, but not participation in another outcome-based interventional trial.

Hence, the administration of a placebo in addition to best supportive care will be used in the control group.

### Intervention description {11a}

All study medication is provided as a kind gift of Zentiva, ltd. Unblinded study pharmacist will prepare 40 ml of sterile water study medication, according to patient’s allocation into the treatment arm:

- Hydroxychloroquine sulphate 200 mg
- Azithromycin dihydrate 500 mg
- Lactose as placebo

For possible drug-drug interactions and details of possible adverse effects, see Summary of Product Characteristics for both investigational medicinal products.

Treatment group allocation is as follows (Table 1):

**Table 1:** Treatment allocation in 3 study arms

Study group		
HC-A (Intervention)	Azithromycin	Hydroxychloroquine
HC (ACTIVE COMPARATOR)	Lactose	Hydroxychloroquine
C (PLACEBO)	Lactose	Lactose

Each study subject will be given:

- Day 1: Patients receive two doses 400 mg of hydroxychloroquine or placebo in 12 h interval and 500 mg of azithromycin or placebo once in 24 h (with the first dose of hydroxychloroquine or placebo)
- Days 2–5: Patients receive two doses 200 mg of hydroxychloroquine or placebo in 12 h interval 250 mg of azithromycin or placebo once in 24 h (with the first dose of hydroxychloroquine or placebo)

#### Masking

The study drugs will be administered in covered Jeannete syringe into nasogastric tube (for patients unable to swallow) or drunk from a black mug by patients who are able to. In both cases, at least 50 ml of water will be used for flushing.

### Criteria for discontinuing or modifying allocated interventions {11b}

In general, the study subject will be withdrawn from the study if any of the following criteria are fulfilled:

- Consent withdrawal by study subject
- In case—in the opinion of the investigator—the study procedures are considered unsafe for the study subject or the risks outweigh the potential benefits

Specifically, for the study subjects enrolled into the study as COVID-19 suspected but without the definite result of rt-PCR testing:

- If COVID-19 is negative and believed to be true negative, subjects will be immediately withdrawn from the study and administration study medication will immediately be stopped. Yet, data on adverse events will still be collected and analysed, in accordance with “intention-to-treat” mode of safety analysis

Note: In case there is a strong suspicion of COVID-19 despite negative test or if there is a doubt about the validity of the test (e.g., questionable sampling technique), it is possible and recommended to repeat the test and only withdraw the subject from the study after the confirmatory test is negative.

### Strategies to improve adherence to interventions {11c}

Adjusting IP administration to patient’s swallowing capability and GI function: During the study, we expect following patients may be enrolled. Those conscious and able to swallow will be given study medication in black mug to swallow. Patients unable to swallow with a nasogastric tube in place will be given the IP via the NG tube reconstituted in sterile water and flushed as per local NG medication guideline. Administration of the IP is temporarily omitted in patients who are unable to swallow but without NG access or do not tolerate any enteral intake (such as patients in profound shock). As soon as the condition preventing IP administration is eliminated, resumption of study medication follows the guidance for day 1.

The role of a dedicated unblinded study pharmacist in each centre is not only to prepare and mask the study drub, but also to ensure its timely administration. If, for any reason, the dose of study drug is omitted, the reason will be documented and appropriate reloading procedure applied for the next dose.

### Relevant concomitant care permitted or prohibited during the trial {11d}

There is no study-specific restriction apart from open-label use of hydroxychloroquine and macrolide antibiotics. All drugs known to prolong QTc must be used with caution. The standard of care also may change in time during the course of the study, for example, if new evidence emerges and changes the state-of-the art treatment recommendations. Any such event will trigger emergency steering committee meeting, and decision will be taken about further action. Research Ethics Board (REB) and regulatory authorities will be notified immediately about the decision taken (see below).

### Provisions for post-trial care {30}

During and after 5 days of study drug administration as well as during the rest of 90 days of follow up, trial participants in all treatment arms will be receiving standard of care, delivered by teams not directly involved in the study and not influenced by their participation in the trial.

### Outcomes {12}

#### Primary outcome

- Composite percentage of patients alive and not on end-of-life pathway who are free of mechanical ventilation at day 14 (see detailed explanation below)

#### Secondary outcomes

- Composite percentage of patients alive and not on end-of-life pathway who are free of mechanical ventilation at day 14 in the subgroup of patients without the need of mechanical ventilation at baseline
- Length of stay in intensive care unit
- Mortality at day 28 and 90

#### Tertiary (exploratory) outcomes

- Viral load at day 8 (no. of viral RNA copies/millilitre of blood)
- Proportion of patients alive and with negative rtPCR nasal swab at D14
- Difference of fraction of inspired oxygen (FiO_2_) between days 0 and 7.
- Difference of respiratory system compliance between days 0 and 7.
- Pharmacokinetic profiles of investigational drugs after oral versus nasogastric administration

Comment to primary outcome: Because the biological plausibility of this trial is in the possibility that hydroxychloroquine inhibits virus replication and azithromycin potentially enhances this, the primary outcome was chosen to prove or disprove that decrease virus replication reduces the damage to the lungs and hence less patients die or progress into frank acquired respiratory distress syndrome (ARDS) with the need of protracted mechanical ventilation and often resulting in delayed death or failed functional outcomes [10].

Mechanical ventilation is defined as the need at any time during the last 24 h of positive pressure ventilation, shall it be ventilation via endotracheal tube, tracheostomy or non-invasive ventilation delivered by a tightly fitting mask. On the contrary, high-flow nasal oxygen support, positive airway pressure via a helmet or uninterrupted spontaneous ventilation via tracheostomy (including with the use of Ayre T-piece with or without positive end-expiratory pressure ventil) is not considered mechanical ventilation. End-of-life care is defined as compassionate care in patients nearing end-of life. Isolated do-not-resuscitate or do-not-reintubate decisions or other ceilings of treatments are not considered end-of-life care, provided that decision is made to continue current level of treatment and actively support patient’s improvements.

### Participant timeline {13}

**Fig. 1:**
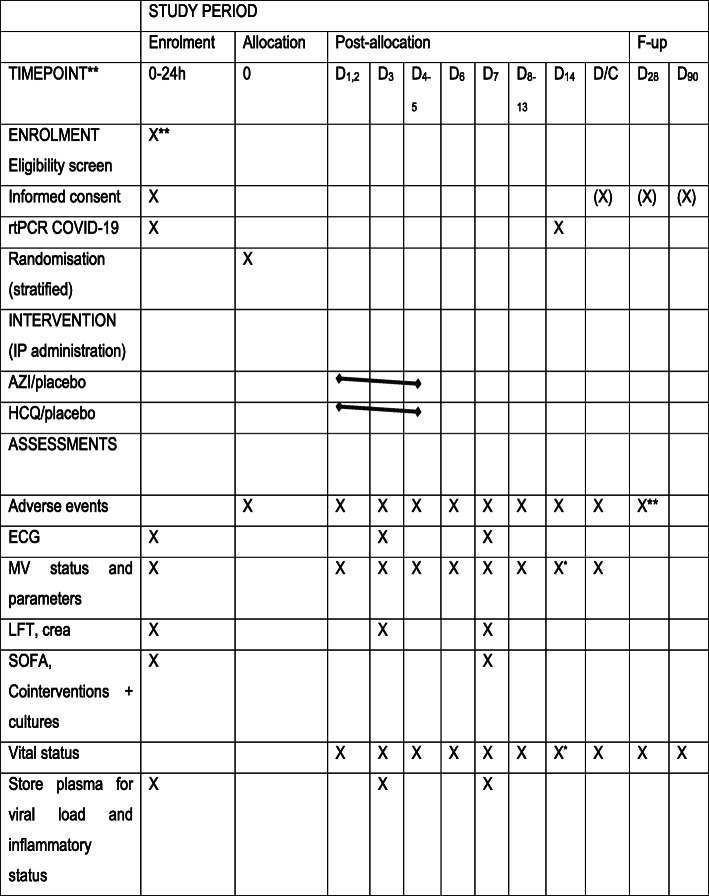
Overview of study procedures. Follow-up can be performed over the phone in outpatients. Note: D/C, discharge from ICU; F-up, follow up; LFT, liver function tests; MV, mechanical ventilation; SOFA, Sequential Organ Failure Assessment. *Primary outcome = being alive and off MV at D14. **This includes pregnancy test if applicable

The patient will have study visits daily as long as he/she stays on ICU or until day 14, whichever occurs earlier. Primary outcome will be recorded at day 14 after enrolment, in hospitalised patients during study visit, in discharged patients over the phone. At discharge from ICU (which may occur before or after day 14) as summary complications and concomitant treatments will be recorded in eCRF. Study subjects will be followed up at days 28 and 90. Details of study procedures are summarised in Fig. 1.

### Sample size {14}

Based on data from Wuhan [6] and Washington [7], where 67% of patients had died within 2 weeks and half of the survivors needed protracted mechanical ventilation, we assume the incidence of the primary outcome in the control group (i.e., being alive and off the ventilator in 2 weeks) to be 25%. The study gives us 80% chance to detect the increase of the primary outcome to 50% in one or both interventional groups at *p* < 0.017 with 74 patients in each arm. In order to compensate for drop-out and loss of follow up, we plan to enrol 240 subjects into the study. No replacement of subjects who dropped out from the study is planned.

### Recruitment {15}

In each study site, a dedicated investigator with the help of the centre coordinator will identify eligible patients every day during morning rounds with the aim to enrol all eligible patients. The central study coordinator will ensure resources are available in all centres at all times including surges of patients during local infection outbreaks.

## Assignment of interventions: allocation

### Sequence generation {16a}

Randomisation procedure will be performed in blocks of 6 and stratified for study centre and age (> 70 or ≤ 70 years). Electronic Case Record Form (eCRF) will perform randomisation using random sequence script in software R and generate medication code. Rationale: Patient’s age is the single most powerful predictor of outcome and stratifying randomisation and stratification decreases the probability of treatment groups being of different age at baseline by chance. In analogy, study centres may vary in the use of non-protocolised treatments, which could bias the results. Randomisation blocking and stratification are described in the protocol and hence will not be concealed for study site investigators, but the computer-generated randomisation sequence will be concealed for all study personnel but the statistician (PW). This prevents any predictability of treatment allocation for subjects before randomisation.

### Concealment mechanism {16b}

Only the unblinded member of the research staff will perform the randomisation procedure. The interface of eCRF will conceal subject treatment allocation to all other personnel involved in the study. Subject allocation report will be placed in an opaque sealed envelope into patients’ medical notes in care emergency unblinding is needed.

### Implementation {16c}

All newly admitted patients in each participating ICU will be screened daily for eligibility and recorded in a screening log in eCRF. All potentially eligible patients or their representatives will be approached by the site investigator and offered participation in the trial. Those consenting will be reported back to the unblinded member of research staff who will perform randomisation procedure and record baseline data in eCRF. All data will be recorded by site investigators into patients’ medical chart and by research nurses into a custom-built eCRF. Follow-up visits or phone calls will be performed by research nurses.

## Assignment of interventions: blinding

### Who will be blinded {17a}

The study medication will be prepared in each centre also by a dedicated unblinded member of research staff and handed over to the treatment team in an opaque syringe or mug, respectively, depending on whether the study medication is to be administered by mouth or into a nasogastric tube. Site investigators, research nurses and coordinators will be blinded to subjects’ treatment allocation. Study statistician will have access to subject treatment codes, but for the interim analysis, he will present processed data only labelled as “group X”, “group Y”, and “group Y”, without disclosing actual treatment allocation. The steering committee can ask for group unblinding if deemed necessary for the subject safety. Both REB and Czech Drug Agency (SUKL) will be notified about the decision to unblind groups in the report letters from interim analysis.

### Procedure for unblinding if needed {17b}

In case the investigator suspects that an adverse event had occurred that represents a threat to the patient’s safety, it is possible to unblind the study treatment allocation. An unblinded pharmacist will insert a card with patient’s treatment allocation into an opaque sealed envelope labelled “Emergency unblinding only, do not open” into the patients’ medical chart. The Principal Investigator must be notified about the use of emergency unblinding procedure within 24 h.

## Data collection and management

### Plans for assessment and collection of outcomes {18a}

Prior to initiation of each study site, the central research coordinator will train the local dedicated study personnel in the use of e-CRF and other study procedures. The medication will be delivered via a central pharmacy in sealed numbered boxes and stored in ICUs. Adhering to Good Clinical Practice rules and guidelines is of upmost importance despite all the challenges during the current pandemic situation. Nonetheless, the eCRF has been designed to balance the safe conduct of the clinical trial and feasibility of timely data entry during staff shortages and overload. In order to do so, all data that is not essential for safety can be input in retrospect. Data on interventions that we know/think influence survival will be monitored and described. This includes concomitant antiviral antimicrobial diagnosis. We will also collect data on how the respiratory specimen for rtPCR diagnostic was collected (smear, endotracheal aspirate, bronchoalveolar lavage). It is likely that most patients will have complete per-protocol data sets whilst in ICU as in critically ill patients most data are recorded as a part or routine care.

### Plans to promote participant retention and complete follow-up {18b}

Day 28 and 90 follow-ups will be performed as physical visit in hospitalised patients or a telephone interview. Vital status, mechanical ventilation status and whether the patient is still in ICU and in hospital are all very robust data that can be obtained with minimum equipoise even over the phone. In addition, the protocol recommends that patient’s self-reported data are checked against hospital notes and/or clinical information system.

### Data management {19} and confidentiality {27}

All data will be stored in password-protected, custom-programmed Oracle-based database linked to eCRF and stored and backed-up in secured servers located within hospital premises. Primary imputation of data into the eCRF will be performed by investigators or a dedicated study personnel under the supervision of investigators. Investigators vouch for source data quality and integrity.

For the sake of all analyses as well as for storage of raw data in a public database, record-level patients’ data will be de-identified as per local legislation.

### Plans for collection, laboratory evaluation and storage of biological specimens for genetic or molecular analysis in this trial/future use {33}

Due to the pragmatic nature of the study, laboratory investigation is minimised to a minimum required for assessing safety. In all patients, only 3 plasma samples will be stored for exploratory analyses described above. We plan to dispose all samples after the analysis. No molecular analyses of human DNA are planned.

## Statistical methods

### Statistical methods for primary and secondary outcomes {20a}

The proportion of patients alive and off mechanical ventilation (primary outcome) between intervention and control groups will be calculated using chi-square test. *p* value is adjusted for multiple comparisons to *p* = 0.017 as there are 3 comparisons between 3 study arms. Survival and ICU/length of stay will be compared using Kaplan-Maier’s curves and exploratory analyses my multi-level regression using statistical packages in software in R. We plan to analyse the primary outcome separately in patients who require mechanical ventilation at baseline from those who do not and we in patients above or below 70 years of age as a priori subgroup analyses.

### Interim analyses {21b}

We plan to perform an interim analysis after the primary outcome is known for the 120th subject. As mentioned above, primary and secondary outcomes will only be calculated in patients with confirmed COVID-19 infection who received at least one dose of IP. Adverse events will be calculated in all randomised patients (intention-to-treat analysis).

All suspected unexpected serious adverse events (SUSARs) will be reported to both REB and to the steering committee, who may decide to stop the trial for safety concerns. Apart from safety, other reasons for stopping the trial are as follows:

1. Emergence of new data (e.g., publication of the results of a big RCT) that may lead the continuation of the trial unethical. This may be due to safety concerns of the placebo group (in case a strong clinical benefit is proven by other trials) or any of the interventional groups (e.g., if harm is reported by other trials). This rule also applies for emergence of a new treatment.
2. After interim analysis: The steering committee will review primary outcomes and the summary of adverse events in all 3 groups (labelled as A, B, and C) whilst still blinded to treatment allocation. The treatment can be stopped if the following criteria are fulfilled:

- There is a significant difference in the primary outcome at *p* < 0.017 in between the groups.
- Futility: The futility criterion is not binding for the steering committee. Based on available data, the study statistician calculates the probability of being able to prove the null hypothesis with achieving the target number of subjects and the probability of type II error made by stopping the trial prematurely.
- Safety: In case the number of adverse events in one or more treatment groups is found uneven or unacceptably high.
- Details of pharmacovigilance are described in a dedicated section below.

### Methods for additional analyses (e.g., subgroup analyses) {20b}

We plan to analyse the primary outcome separately in patients who require mechanical ventilation at baseline from those who do not and we in patients above or below 70 years of age as a priori subgroup analyses.

### Methods in analysis to handle protocol non-adherence and any statistical methods to handle missing data {20c}

Primary and secondary outcomes will be calculated and reported for all patients that were found to have COVID-19 infection at enrolment and received at least one dose of study medication or placebo, whilst those who were randomised based on clinical suspicion and later found not to have COVID-19 will be excluded (per-protocol analysis). Adverse events and safety outcomes will be reported as intention-to-treat analysis. No replacement of study subjects or imputation of missing data will be made.

### Plans to give access to the full protocol, participant level-data and statistical code {31c}

Full protocol is available on the website www.donatio-intensivistam.cz and de-identified participant-level dataset will be made available 6 months after the publication of the results of the study at www.mendeley.com.

## Oversight and monitoring

### Composition of the coordinating centre and trial steering committee {5d}

The steering committee consists of the principle investigator, one investigator representative from each study site, lead study pharmacist, research methodologist, study statistician and patients’ representative. Non-voting members are the central study coordinator and legal advisor. The main role of the steering committee is overall supervision of the trial; the committee meets at least biannually, to review all reported SUSARs as well as new evidence in the field of COVID-19 treatment. The committee makes by majority vote the final decision as to whether to stop or continue the trial after interim analysis or should any safety concerns arise. The principle investigator chairs the steering committee and reports to the sponsor and legal authorities such as REB or SUKL.

### Composition of the data monitoring committee, its role and reporting structure {21a}

The funder appointed an independent data monitor. The eCRF is designed to generate queries and reports on missing data or unusual data entries, which will be fed back to the study site investigators in regular intervals.

### Adverse event reporting and harms {22}

The study will be conducted in accordance with the protocol, Good Clinical Practice and current Czech legislation. It is of upmost importance for the safety of all subject in this and other trials that all personnel involved in this trials complies with the KLH-21, version 7, directive of SUKL on Reporting Adverse Reactions to Medicinal Products for Human Use in a Clinical Trial and to Medicinal Products without Marketing Authorisation, issued in accordance with the provisions of Section 56, point 13 Act No 378/2007 Coll., on Pharmaceuticals and on Amendments to Some Related Acts, as amended. In particular, the investigator is obliged to promptly report all SUSARs to the sponsor. A prompt report (no later than within 24 h from the moment when the investigator has learnt about the fact) will be followed by detailed written reports. In prompt and follow-up reports, subjects are identified using unique numerical codes, which were assigned to the subjects.

Of note, this clinical trial is performed on a population of critically ill patients with a very high mortality rate. Therefore, if the death of a study subject is deemed by the investigator as resulting from the natural course of the underlying disease and no relation to study treatment or procedures is suspected, such an event shall not be considered SAE and reported as such.

### Frequency and plans for auditing trial conduct {23}

The trial may be subject of an audit from regulatory authorities such as SUKL

### Plans for communicating important protocol amendments to relevant parties (e.g. trial participants, ethical committees) {25}

Any amendments will be, prior to implementation, submitted for review to both central and local REBs and SUKL. After approval and implementation, they will be registered at www.clinicaltrials.gov.

## Dissemination plans {31a}

We will submit the main results of the trial in an open-access peer-reviewed journal within 3 months after the 240th subject completed the 90-day follow-up visit, which is expected to happen in Q4 of 2021. Results will also be submitted to www.clinicaltrial.gov at the time of publication.

## Discussion

This is an investigator-initiated trial endorsed by the Czech Society of Anaesthesia and Intensive Care. The most significant resource for the study is unpaid voluntary work of all the personnel conducting the study, who decided to do so in times of worldwide pandemic crisis. Part of the resources was gathered from voluntary donors in a crowdfunding campaign conducted by the medical student association. The pharmaceutical company Zentiva ltd. was approached by investigators and kindly agreed to provide study medication at no cost; however, it has had nor will have any role in study design; collection, management, analysis, and interpretation of data; writing of the report; or the decision to submit the report for publication.

This trial investigates two drugs that are being used off label to treat patients with severe COVID-19 with a balance of potential benefits and harms. In vitro and observational trial suggest their potential to limit viral replication and the damage to lungs as the most common reason for ICU admission. Therefore, patients most likely to benefit from the treatment are those with severe, but early disease. The potential benefit is based on biological rationale and observational data [5] showing that one or both drugs may prevent progression of a disease which led to death in 67% of patients in a similar condition to those we plan to randomise [11]. Possible harms include QT prolongation and resulting life-threatening arrhythmias or retinopathy, which may result in blindness. We believe that clinical equipoise regarding risk/benefit to the individual patient justifies well the conduct of this RCT.

We acknowledge that the power of this study is relatively low, as it is only powered to detect 25% difference in the primary outcome. This is why we decided to make de-identified participant-level data available in a public database so they can be used in patient-level meta-analyses should trials with a similar or identical design are conducted anywhere in the world.

## Trial status

RECRUITING

Protocol AZIQUINE-ICU 25032020 Version 1.6, last amended on 22nd June 2020.

Start of recruitment 20th April 2020.

End of recruitment (expected) 30th September 2021.

## Supplementary information

**Additional file 1.** Collaborating institutions/Principal Investigators.

**Additional file 2.** Appendix.

## Abbreviations

GCP: Good Clinical Practice
ICU: Intensive care unit
eCRF: Electronic Care Report Form
FiO_2_: Fraction of oxygen in inspired air
F-up: Follow up
LFT: Liver function tests
MV: Mechanical ventilation
PEEP: Positive end-expiratory pressure
pCO_2_: Partial pressure of carbon dioxide in arterial blood
pO_2_: Partial pressure of oxygen in arterial blood
REB: Research Ethics Board [Etická komise]
SOFA: Sequential Organ Failure Assessment
SUKL: Czech Drug Control Office [Státní ústav pro kontrolu léčiv]
SUSAR: Suspected unexpected serious adverse event
