# Supplementary material for: Azithromycin added to hydroxychloroquine for patients admitted to intensive care due to coronavirus disease 2019 (COVID-19)—protocol of randomised controlled trial AZIQUINE-ICU
Source: Trials. 2020 Jul 8;21:631. doi: 10.1186/s13063-020-04566-x (PMC7341702; doi:10.1186/s13063-020-04566-x)
Supplement: Supplementary file 1 — Additional file 1. Collaborating institutions/Principal Investigators. [file 13063_2020_4566_MOESM1_ESM.docx]

COLLABORATING INSTITUTIONS/Principal Investigators

| Fakultní nemocnice Královské Vinohrady, Klinika anesteziologie a resuscitace, Šrobárova 1150/50, 100 34 Praha 10 / doc MUDr. František Duška, PhD. | Šrobárova 50, 100 34 Praha 10 |
| --- | --- |
| Všeobecná fakultní nemocnice v Praze, Klinika anesteziologie a resuscitace, doc. MUDr. Martin Balík, PhD. | Na Bojišti 1, 128 08 Praha 2 |
| Fakultní nemocnice Motol, Klinika anesteziologie, resuscitace a intenzívní medicíny, doc. MUDr. Tomáš Vymazal, Ph.D., MHA | V úvalu 84, 150 06, Praha 5 |
| Fakultní nemocnice u sv. Anny v Brně, Anesteziologicko-resuscitační klinika, MUDr. Václav Zvoníček, PhD. | Pekařská 53, 656 91 Brno |
| Fakultní nemocnice v Plzni, Klinika anesteziologie, resuscitace a intenzívní medicíny, doc. MUDr. Jan Beneš, Ph.D. | Edvarda Beneše 1128/13  305 99 Plzeň-Bory |
| Krajská zdravotní, a.s. - Masarykova nemocnice v Ústí nad Labem, o.z., Klinika anesteziologie, resuscitace a intenzívní medicíny, prof. MUDr. Vladimír Černý, PhD | Sociální péče 3316 /12A, 400 11 Ústí nad Labem |
| Fakultní nemocnice Olomouc, Klinika anesteziologie, resuscitace a intenzívní medicíny, MUDr. Olga Klementová, PhD. | I. P. Pavlova 185/6, 779 00 Olomouc |
| Nemocnice Na Bulovce, Anesteziologicko-resuscitační oddělení, Prim. MUDr. Jakub Bala | Budínova 67/2. Praha 8 – Libeň 180 81 |
