# Supplementary material for: Azithromycin added to hydroxychloroquine for patients admitted to intensive care due to coronavirus disease 2019 (COVID-19)—protocol of randomised controlled trial AZIQUINE-ICU
Source: Trials. 2020 Jul 8;21:631. doi: 10.1186/s13063-020-04566-x (PMC7341702; doi:10.1186/s13063-020-04566-x)
Supplement: Supplementary file 2 — Additional file 2. Appendix. [file 13063_2020_4566_MOESM2_ESM.docx]

Informovaný souhlas s účastí v klinickém hodnocení

**Klinické hodnocení:**

Protokol: AZIQUINE-ICU 25032020

Eudra CT number: 2020-001456-18 Registrační číslo NCT04339816

**Název:**

AZIQUINE-ICU, Azitromycin přidaný k hydroxychlorochinu v léčbě pacientů přijatých do intenzívní péče s infekcí COVID-19 - randomizovaná kontrolovaná studie (fáze 3)

**Sponzor:** Nadační fond Donatio Intensivistam: www.donatio-intensivistam.cz

**Zkoušející lékař:**

Vážená paní, vážený pane,

dovolujeme si Vám nabídnout účast v klinickém hodnocení léčiv – protože se jedná o výzkumnou činnost, je k tomu potřeba Váš souhlas.

**Úvod – stručné představení studie**

V současné době probíhá celosvětová pandemie koronavirem SARS-CoV-2 způsobujícím onemocnění COVID-19. Většina onemocnění má lehký průběh, ale menšina nemocných vyžaduje hospitalizaci a několik procent nakažených umírá. Nejčastější příčinou hospitalizace je ztížené dýchání v důsledku postižení plic virózou. Příčinnou léčbu této infekce zatím neznáme, a proto je současným standardem léčby terapie podpůrná – spočívající většinou v podpoře dýchání podáním kyslíku nebo umělou plicní ventilací a v indikovaných případech podáním antibiotik jako prevence nasedlé infekce bakteriální. Na buněčných kulturách a prvních datech na pacientech (v kazuistikách a pozorovacích studiích) se zdá, že by dělení viru mohlo být zpomaleno lékem hydroxychlorochin přípravek Plaquenil, který se používá v léčbě malárie nebo některých autoimunitních nemocí) nebo jeho kombinací s dlouho užívaným antibiotikem azitromycinem (Azitrox, Zitromax). Oba tyto léky jsou již po léta registrované a v jiných indikacích používané, ale jejich účinnost na infekci virem COVID-19 není známa.

Ve studii, v níž Vám nabízíme účast, budou pacienti, kteří byli z důvodu těžkého průběhu infekce COVID-19 přijati do intenzívní péče, náhodně vybráni v poměru 1:1:1 do 3 skupin. U všech pacientů bude probíhat standardní podpůrná léčba, jak je popsána výše. Navíc jedna skupina bude dostávat po 5 dní kombinaci hydroxychlorochinu a azitromycinu, druhá hydroxychlorochinu a stejně vypadající neúčinné látky (placeba) a třetí skupina bude léčena pouze placebem (a standardní podpůrnou léčbou). Hlavním vyhodnocovaným měřítkem je počet pacientů, kteří jsou naživu a nepotřebují umělou plicní ventilaci po 14 dnech od zařazení do studie. Dále budou sledovány nežádoucí účinky, které tato léčba může způsobit, délka pobytu na jednotce intenzívní péče a vylučování viru do tělních tekutin po dvou týdnech od zařazení do studie a přežití pacientů po 28 a 90 dnech.

O své účasti ve studii se můžete poradit se svými blízkými a vyžádat si dostatek času k prostudování dokumentu. Klinické hodnocení je organizováno Českou společností anesteziologie, resuscitace a intenzívní medicíny, má nekomerční charakter. Studijní léky pro studii zdarma poskytla firma Zentiva, a.s.

**Charakteristika hodnocených léčivých přípravků**

Hydrochlorochin je látka, která se po mnoho desetiletí užívá v léčbě autoimunitních chorob jako např. revmatoidní artritida a malárie. Zdá se, že by hydroxychlorochin mohl tlumit množení viru v buňce a zmírňovat průběh nemoci. Z nežádoucích účinků jsou nejvýznamnější změny elektrické srdeční aktivity (měřené pomocí EKG, elektrokardiogramu), které vzácně mohou vést k poruchám srdečního rytmu a velmi vzácně byly popsány změny na sítnici. Azitromycin je látka užívaná jako antibiotikum, která brání přepisu mikrobiálních ribonukleové kyseliny do molekuly bílkovin (translaci) a existují data z pozorovacích studií o významném snížení virové nálože u pacientů užívajících tuto látku. Nejčastějším nežádoucím účinkem azitromycinu je přechodné zvýšení jaterních testů (laboratorních známek poškození jater).

**Proč studii děláme?**

Cílem klinického hodnocení je zhodnotit, jak hydroxychlorochin samostatně anebo v kombinaci s azitromycinem působí na průběh nemoci COVID-19. Nevíme, zda je lék účinný a zda je bezpečný. Účelem studie je to zjistit.

**Jaká je pravděpodobnost, že dostanu účinnou látku a jak studie probíhá?**

Pacienti se do léčebných skupin náhodně rozdělují v poměru 1:1:1, tzn. máte 67% pravděpodobnost, že budete léčen(a) hydroxychlorochinem a 33% pravděpodobnost, že budete dostávat i azitromycin a 33% pravděpodobnost, že budete dostávat pouze placebo. Placebo je přípravek, který neobsahuje žádnou účinnou látku. Po dobu 5 dnů budete dostávat 2x denně 2 tablety, které budou obsahovat hodnocená léčiva nebo neúčinnou látku (placebo). Všechny ostatní aspekty léčby vč. léčby podpůrné, podpory a náhrady funkcí selhávajících orgánů nejsou nijak omezeny a budou probíhat stejně mezi léčebnými skupinami jako v případě, že byste do studie zařazen(a) nebyl(a).

**Postupy a výkony v průběhu klinického hodnocení**

V průběhu léčby bude Váš zdravotní stav pečlivě monitorován jako u pacientů mimo studii. Navíc se 0., 3. a 7. den provedou odběry cca 20 ml krve a natočí se EKG křivka. Hospitalizace bude probíhat tak dlouho, jak je to nutné (účast ve studii jí nijak neprodlouží). V případě propuštění budete navíc 28. a 90. den kontaktováni telefonicky studijní sestrou. V případě hospitalizace bude prováděn 14. den kontrolní stěr z nosohltanu pomocí tyčinky ke zjištění přítomnosti viru. Předpokládané množství celkově odebrané krve je cca 60 ml v celém průběhu studie.

**Odpovědnost účastníka hodnocení**

Pokud souhlasíte s účastí v klinickém hodnocení, zavazuje Vás to k dodržování pokynů zkoušejícího lékaře a studijního týmu. O jakékoliv jiné léčbě mimo studijní medikaci, kterou byste chtěl(a) po propuštění užívat, byste se měl(a) předem poradit se zkoušejícím lékařem. Platí to i o volně prodejných lécích a doplňcích stravy. Při léčbě hodnoceným přípravkem se po nějakou dobu rovněž nesmíte slunit.

**Co pro mě účast na výzkumu znamená?**

Vaše léčba se v průběhu studie bude lišit v tom, že za Vámi přijde 3x studijní sestra k natočení EKG a provedení odběrů krve – pokud budou tyto indikovány z nestudijních důvodů, provedou se zároveň. Rovněž telefonická kontrola studijní sestrou 28. a 90. den je čistě studijní procedurou.

**Předvídatelná rizika či nepříjemnosti pro účastníka hodnocení**

Patří mezi ně 3x odběr krve a možný výskyt nežádoucích účinků zkoumaných látek: předvídatelných zmíněných výše, i nepředvídatelných. Účinek studovaných léčiv není znám, a proto je možné, že Váš zdravotní stav nemusí zlepšit nebo jej dokonce horší, např. díky výskytu nežádoucích účinků. V případě výskytu nežádoucích účinků i po propuštění do domácí péče byste měl(a) hned kontaktovat zkoušejícího lékaře.

Rizika pro plod nebo kojené dítě, antikoncepce: Ze studie jsou nezletilí i těhotné ženy vyřazeny. Podávání studijních látek bude prováděno na jednotce intenzívní péče, kde je předpokládána sexuální abstinence. Těhotenský test u žen ve fertilním věku bude proveden v den přijetí a poté po měsíci.

**Očekávané přínosy**

Je možné, že hydroxychlorochin samostatně nebo v kombinaci s azitromycinem zmírní průběh onemocnění COVID-19 a některým pacientům umožní vyhnout se potřebě napojení na umělou plicní ventilaci.

**Jiné dostupné možnosti léčby**

V čase hodnocení není v ČR dostupná žádná prověřená a schválená účinná látka ke příčinné (kauzální) léčbě onemocnění COVID-19.

**Podmínky odškodnění** v případě újmy na zdraví vzniklé v souvislosti s účastí v klinickém hodnocení. Všichni účastníci klinického hodnocení jsou pojištěni podle platné legislativy v ČR. V případě újmy na zdraví v důsledku účasti ve studii se obraťte na zkoušejícího lékaře. Bude Vám zajištěna a uhrazena odpovídající lékařská péče, a máte právo na odškodnění v souladu s platnými právními předpisy. Podpisem tohoto informovaného souhlasu se nevzdáváte žádného ze svých zákonných práv.

**Dostanu za účast ve studii nějakou odměnu?**

Pokud by Vám v souvislosti se studií vznikly nějaké náklady (cestovné, telefonní poplatky, ušlá mzda), budou Vám uhrazeny. Za samotnou účast ve studii žádná odměna není.

**Účast subjektu hodnocení je dobrovolná**

Svou účast ve studii můžete svobodně odmítnout, a to kdykoliv a bez udání důvodu, a přitom tím vztah s lékařem a Vaše léčba nebudou ovlivněny. Přesto Vám doporučujeme úmysl odstoupit od studie vždy konzultovat s Vaším lékařem.

P**řístup ke zdravotní dokumentaci a uchovávání informací**

Veškeré záznamy budou uchovávány a bude s nimi nakládáno v souladu s platnou legislativou České republiky, včetně Obecného nařízení o ochraně osobních údajů (GDPR). Sdělení ke zpracování osobních údajů je samostatným dokumentem, který obdržíte.

Všechny údaje a odebrané vzorky získané v klinickém hodnocení budou odeslány zadavateli pouze v kódované podobě. Údaje umožňující zjistit Vaši totožnost tak neopustí pracoviště zkoušejícího lékaře. Do Vašich osobních záznamů ve zdravotnické dokumentaci má přístup pouze zkoušející lékař, pověření zástupci zadavatele (např. monitor a auditoři), osoby pověřené národními kontrolními úřady a členové etické komise, tzn. osoby pověřené dohledem nad průběhem klinického hodnocení. Tyto osoby jsou vázány povinnou mlčenlivostí.

**Co když se objeví převratná nová informace?**

Pokud se vyskytne informace, která by mohla mít význam pro Vaše rozhodnutí pokračovat v účasti v klinickém hodnocení, budete ihned informován(a). Informace Vám budou sděleny neprodleně především v případě, že by se jednalo o vliv na Vaší bezpečnost.

**Doba trvání studie a předvídatelné okolnosti a důvody, pro které může být Vaše účast v klinickém hodnocení ukončena**

Podávání látky se provádí po dobu 5 dnů, studie sama končí telefonátem 90. den po zařazení. Vaše účast může být předčasně ukončena, pokud se objeví pochybnost o bezpečnosti zkoumané látky nebo pokud byste nedodržoval(a) studijní postupy. Přibližný́ počet subjektů, které se budou klinického hodnocení účastnit v České republice je 240. O Vaší účasti v klinickém hodnocení bude informován Váš praktický lékař (v souladu požadavkem přílohy č. 2 vyhlášky o SKP a zákonem o zdravotních službách - § 45 práva a povinnosti poskytovatele, odst. (2): "Poskytovatel je povinen předat zprávu o poskytovaných zdravotních službách registrujícímu poskytovateli v oboru všeobecné praktické lékařství, anebo v oboru praktické lékařství pro děti a dorost").

**Kontakt na osoby, od kterých může účastník klinického hodnocení obdržet další informace**

V případě, že byste kdykoli potřeboval(a) další informace, neváhejte se obrátit na hlavního zkoušejícího:

doc. MUDr. František Duška, PhD. email: [frantisek.duska@lf3.cuni.cz](mailto:frantisek.duska@lf3.cuni.cz), tel. 608405541

Ohledně etických aspektů a práv jeho účastníků se můžete obrátit na tyto etické komise, které schválily průběh studie:

Multicentrická etická komise Královské Vinohrady, Šrobárova 1150/50, Praha 10, 100 34,

Předseda EK FNKV Prof. MUDr. Jan Pachl, CSc. ,Tel.: +420 267 16 2987,

E-mail: jan.pachl@fnkv.cz

Lokální etická komise – název, adresa, kontakty:

**Svým podpisem stvrzuji, že jsem výše uvedeným informacím porozuměl(a), měl(a) jsem možnost položit ke studii otázky, které mi byly uspokojivým způsobem zodpovězeny a že na základě dávám souhlas se svou účastí ve studii.**

• **Účastník klinického hodnocení (pacient)**

| Datum  (formát DD/MM/RRRR) | Jméno účastníka hůlkovým písmem | Podpis účastníka |
| --- | --- | --- |
|  |  |  |
| ………………………………… | …………………………………………………… | ………………………………… |

• **Zkoušející lékař**

Po podpisu účastníka hodnocení i zkoušejícího obdrží účastník klinického hodnocení stejnopis dokumentu (ne kopii).

| Datum  (formát DD/MM/RRRR) | Jméno zkoušejícího lékaře hůlkovým písmem | Podpis zkoušejícího lékaře |
| --- | --- | --- |
|  |  |  |
| ………………………………… | …………………………………………………… | ………………………………… |

• **Nezávislý svědek:** Pokud jsou informace účastníkovi přečteny z důvodu poruchy zraku nebo pokud účastník dává informovaný souhlas ústně, protože není schopen podpisu, je třeba nezávislého svědka, který se podepíše zde:

| Datum  (formát DD/MM/RRRR) | Jméno nezávislého svědka hůlkovým písmem | Podpis nezávislého svědka |
| --- | --- | --- |
|  |  |  |
| ………………………………… | …………………………………………………… | ………………………………… |

PŘÍLOHA – PACIENTI V BEZVĚDOMÍ NEBO BEZ SCHOPNOSTI UDĚLIT INFORMOVANÝ SOUHLAS:

Potvrzuji tímto, v souladu se Zákonem o léčivech, § 52 odst. (9), že pacient není schopen udělit informovaný souhlas, jeho zákonný zástupce není stanoven nebo je nedosažitelný. Pacient splnil vstupní kritéria zařazení do studie

• **Nezávislý lékař**

| Datum  (formát DD/MM/RRRR) | Jméno nezávislého lékaře hůlkovým písmem | Podpis nezávislého lékaře |
| --- | --- | --- |
|  |  |  |
| ………………………………… | …………………………………………………… | ………………………………… |

Svým podpisem potvrzuji, že byl subjekt hodnocení zařazen v souladu s postupem popsaným v protokolu a schváleným etickou komisí

• **Svědek**

| Datum  (formát DD/MM/RRRR) | Jméno svědka hůlkovým písmem | Podpis svědka |
| --- | --- | --- |
|  |  |  |
| ………………………………… | …………………………………………………… | ………………………………… |
